# Lives that Matter: Criminology and Global Security Inequality

**DOI:** 10.1007/s43576-021-00007-0

**Published:** 2021-03-05

**Authors:** Katja Franko

**Affiliations:** Department of Criminology and Sociology of Law, University of Oslo, Oslo, Norway

**Keywords:** Inequality, Globalization, Citizenship, Race, Sovereignty

## Abstract

Ideals of universalism and the idea that all lives are equally valuable, and should be equally worthy of protection, form a standard narrative for human rights regimes and international legal instruments. However, realities on the ground are marked by social arrangements where lives are de facto unequally protected. The article addresses one of the central criminological concepts and perspectives—that of inequality—and examines possible avenues for theorizing the dynamics of global security inequality. How well equipped is criminology to address the underlying processes of social stratification which shape decisions about whose life gets to be protected, and by what means? What might a global understanding of inequality look like? To what extent do existing concepts for analyzing inequality enhance or impede our understanding of global social cleavages?

The Norwegian coastal city of Bergen is home to less than 300,000 inhabitants. In August 2020, the local police issued a statement expressing frustration about the deteriorating relations with ethnic minority youth. According to a police unit leader, they could notice a change after the summer’s highly publicized Black Lives Matter protests in the United States: “We are being portrayed as a very violent organization, one that is not on the same side as the citizens and which discriminates against certain ethnic and age groups.” Also the National Police Commissioner issued a statement saying that Norwegian youth should not draw parallels with how police in the US do their job: “These are quite different countries with different cultures and different understanding of the role of the police.”^1^ Although their analysis was critiqued in the subsequent public discussion that pointed out a more complex background and a number of local conditions behind the developments, the statements are, nevertheless, revealing of an interesting social dynamic. They point to how globally visible events weigh heavily on the minds of seemingly remote local actors. Internationally, concerns about biased policing have resonated across a variety of jurisdictions and have led to numerous protests, which can be seen as a form of global consciousness or even a global justice movement (Della Porta, 2016).

However, the relationship between global connectivity and global consciousness is, as Roland Robertson (2011) points out, by no means simple and straight forward. The latter does not derive directly from the former but has a certain degree of autonomy. At a time when global forces are in many ways on the retreat—and localism and the national seem to have “won” through the bio-political necessity imposed by the current pandemic—the distinction between global connectivity and consciousness makes a useful starting point for a discussion on the future of internationalization in criminology. For while borders are closing, most types of cross-border mobility are declining or brought to a halt, and people are, in practical terms, increasingly confined to their localities, our eyes are on the world. Exemplified by the popularity of the COVID-19 Dashboard operated by Johns Hopkins University, the current situation is marked by intense interconnectedness and common exposure to a cross-border threat, combined with intense focus on national(ism) and local self-preservation in terms of responses. While the tenets of the world risk society may be of a world shrinking due to de-bounding of risks (Beck, 2002), the responses have been often marked by global divisions, self-interest and a lack of solidarity.

What, then, are implications for criminology? In line with the growing intensity of cross-border interconnectedness, the past two decades have seen a remarkable growth of internationally oriented strands of criminology, testified also by the establishment of this journal. However, despite the strength and vigor of these efforts, a powerful line of critique has in recent years pointed out that, whether nationally or internationally oriented, as a discipline, criminology is marked by deep inequalities and divisions, namely, those between the global North and South (Aas, 2012; Agozino, 2003; Carrington et al., 2016; Goyes, 2019; Moosavi, 2019). Internationalization has taken place within a Northern-centric frame, therefore, critics suggest, criminology is ripe for decolonization.

Drawing on these debates, this article addresses the dichotomy between the growing efforts, which are taking the discipline beyond the national frame, and the persistence of global divisions. The article suggests that in order to develop the discipline in a more globally oriented and inclusive direction, the debates need to move beyond issues of representation and touch not only upon *who* does criminology and *where* (although these discussions certainly have merit) but also *how* is criminology done in terms of its conceptual, methodological and theoretical perspectives. A call to examine our analytical toolbox may seem self-evident and in some ways superfluous; it is certainly not new. However, the task, although obvious, demands a fair amount of collective work, which is only in its nascence (see inter alia Carrington et al., 2016; Lohne, 2020; Moosavi, 2019).

The article embarks on this effort by examining one of the central concepts and perspectives in the discipline—that of inequality. How well equipped is criminology to capture and analyze global inequality? What might a global understanding of inequality look like? To what extent do existing concepts for analyzing inequality enhance or impede our understanding of global social cleavages? In particular, the article examines the globally unequal protection of life. Although the right to life is one of the most important human rights, on the ground realities of how it is operationalized vary enormously. Currently, there is a dearth of conceptual and theoretical perspectives for why and how this is so. And while the BLM movement has brought the issue to the attention in terms of the existence of domestic hierarchies of citizenship, a question can be asked about how well equipped are we to analytically capture such dynamics at the global level.

## Criminology and Global Inequality

As Karen Heimer pointed out in her presidential address to the American Society of Criminology, the study of inequalities ‘lies at the heart of our discipline’ (Heimer, 2019, p. 378). Inequality has increased in recent years, in the United States and globally, and has had a negative impact on the legitimacy of social institutions, including criminal justice. Heimer suggests that ‘bringing our social scientific and empirical lenses to bear on the ways that inequalities impact crime can be seen to be a responsibility of the field of criminology’ (ibid.: 380). Criminology should thus have greater focus on intersecting inequalities of class, gender, race and ethnicity, which operate to shape crime, victimization, and criminal justice experiences. Heimer’s intervention is part of a long and productive tradition within criminology on both sides of the Atlantic. In fact, she rightly points out that ‘a focus on inequalities offers an important theoretical thread that stiches our field together into a coherent and strong whole’ (ibid.: 378).

While mentioning global inequality, this body of scholarship, nevertheless, does so mostly in passing, rather than systematically incorporating global perspectives into the analysis. This is not to say that analytical perspectives stemming from global inequality and colonialism have not been applied to studies of crime and criminal justice (see inter alia Gilroy, 1982; Agozino, 2003; Bosworth & Flavin, 2007; Cunneen & Tauri, 2016). Also studies of immigration and ethnicity are one obvious example. Nevertheless, even here the understanding of global dimensions tends to be implicit rather than explicit, leaving the analysis defined by a national framework. As Patil (2013) points out, despite its international mobility, intersectionality is in its origin a domestic concept. Several critical voices have thus argued that globalized dynamics pose challenges for intersectionality and have called for a re-reading of the approach (Henne & Troshynski, 2013; Sanchez, 2017). Feminist critiques of intersectionality, in particular, have argued for a better understanding of how the postcolonial condition demands a transcendence of the traditional race-class-gender nexus and an acknowledgement of global divergences among women (Henne & Troshynski, 2013, p. 463; see also Chatelain & Asoka, 2015).

Also within the field of transnational migration scholars have called for an acknowledgement of the complexity which in important ways challenges the capability of intersectionality’s model (Sanchez, 2017, p. 52). We need not only knowledge on how immigration may lead to social disadvantage, but also a thorough understanding of citizenship as a global regime for distribution of privilege and an analytical apparatus that can challenge the practices of criminalization stemming from it (Bosworth, 2012; Sanchez, 2017; Franko, 2020b). Within the national frame, citizenship is, as Western (2014) points out, ‘a public declaration of equality’, albeit far from complete, as forcefully pointed out by the BLM movement. Within a global frame, however, citizenship functions as a mechanism of social stratification and a system for distribution of privilege (Barker, 2018; Franko, 2020b). For it is precisely the positioning of immigrants within a particular global regime whereby, as Dauvergne (2008) points out, the accident of being born in the global South functions as a legal handicap for citizens of these countries, that has a number of criminal justice implications (Aliverti, 2013; Bosworth et al., 2018). Contemporary cross-border mobility is stratified according to long-standing hierarchies of citizenship (Franko, 2020b), where even the label migrant is, as Basaran and Guild (2017, p. 273) observe, ‘reserved for those associated with particular origins and geographies. Embedded in colonial politics and sustained in postcolonial imaginaries’.

Ulrich Beck’s (2003, p. 50) observation made almost two decades ago that the ‘fragmentation of the world into nation states removes accountability for global inequalities’ remains salient today. In what follows, I will, based on examples from my previous and current research, reflect upon how we might continue these conversations and discuss and examine the possibilities for, empirically and theoretically, teasing out and addressing questions of global inequality. In particular, my focus will be on globally stratified mechanisms for the protection of life. While the BLM movement has forcefully brought to attention biased policing practices and harmful, and often lethal, nature of state interventions over racial and ethnic minorities in the United States, a similar dynamic is also being played out over globally disadvantaged populations. The national and global systems of stratification to some extent overlap but, as I shall proceed to argue, in important ways do not. Understanding global social cleavages demands not only the traditional (national) focus on the intersections of race, class and gender, but also on the inequalities and harmful effects created by a particular world order, in its present and historic forms.

## Global Security Inequality and Hierarchies of Citizenship

Ideals of universalism and the idea that all lives are equally valuable, and should be equally worthy of protection, form a standard narrative for human rights regimes and international legal instruments. The UN Universal Declaration of Human Rights declares in article 3 that ‘Everyone has the right to life, liberty and security of person’. Similarly, article 2.1 of the European Convention of Human Rights declares that ‘Everyone’s right to life shall be protected by law’. However, realities on the ground are marked by social arrangements where, globally, lives are de facto unequally protected. This security inequality is a result of underlying processes of social stratification which shape decisions about which lives are protected, and by what means.

My first insight into this form of inequality came during a project on the European Border and Coast Guard Agency, Frontex (Aas & Gundhus, 2015; Franko, 2020b). Established in 2004, the agency has seen a remarkable growth in budget and tasks and has, according to its self-presentation, transitioned into ‘Europe’s first uniformed law enforcement service’.^2^ This rapid growth encompasses not only its operative capacities and jurisdiction but also its analytical capabilities, which include systematic collection of data and publication of quarterly and yearly reports and statistics on numerous types of border-related activities. However, during our fieldwork we discovered that the agency did not collect data on border deaths, despite the fact that the Mediterranean is arguably the most fatal border in the world and accounts for the vast majority of migrant deaths recorded globally (IOM, 2017, p. 6). We found evidence though that individual officers and Frontex employees saw this as an important issue, some even collected migrant mortality numbers at their own initiative (Aas & Gundhus, 2015).

As pointed out by Andreas and Greenhill (2010, p. 1) “to measure something—or at least claim to do so—is to announce its existence and signal its importance and policy relevance”. While Frontex statistics offer ample substantiation of the threats that migrants are seen to represent to European borders and member states (Franko, 2020b), border deaths remain undocumented by the agency. In 2013, the International Organization of Migration (IOM) started collecting such data through its Missing Migrants Project, while Frontex occasionally refers to these numbers in their reports. The job of counting has thus been outsourced to non-state actors such as IOM and various research projects (Last et al., 2017). Consequently, due to the absence of robust and reliable reporting mechanisms and limited resources,’ data on migrant fatalities are, at best, estimates and at worst, serious undercounts’ (IOM, 2017, p. 21).

According to Nicholas De Genova (2018), the European “migrant crisis” can best be understood as the equivalent of racial oppression in the United States and that it deserves to be framed as an issue of black lives matter. Given that ‘the horrendous risk of border-crossing death systematically generated by the European border regime is disproportionately inflicted upon migrants and refugees from sub-Saharan Africa’, De Genova (ibid.: 1767) argues, this ‘may be taken as the very definition of racism’. By addressing them simply as migrants, scholarly literature is encouraging a process of de-racialization. Several observers have pointed out the reluctance of European scholars to use the category of race (van der Woude, 2020). European identity has been, as Fatima El-Tayeb (2020) points out, shaped by its colonial history as well as amnesia and erasure of these processes from the continent’s collective memory. The reluctance to talk about race is thus problematic (Parmar, 2017). However, it may in certain ways also be analytically more attuned to the European context where ‘race is just one catalyst, among others’ particularly when it comes to crimmigration arrangements (Brandariz, 2020).

The European Union’s treatment of migrant deaths is marked by an institutional logic, which systematically builds on the distinction between the citizen and the alien. Although one might argue that omission to officially count deaths at the border and to identify the dead would seem more problematic to the authorities if the deceased were not racialized, it would certainly be unthinkable if they were EU citizens. Some EU states, such as Greece and Italy, have invested significant efforts toward counting and identification. They are, nevertheless, still unable ‘to identify a significant fraction of the dead’ (IOM, 2017, p. 71). Such administrative knowledge gaps would be unthinkable with regard to the citizen population, which is thoroughly subjected to the Foucauldian bio-political rationalities, where not only death but also numerous other types of ‘loss of vitality’ of the population due to disease and ill health are meticulously measured, aggregated and acted upon (Villadsen & Wahlberg, 2015, p. 10). This line of thinking is clearly also in evidence during the current pandemic.

A question, therefore, needs to be asked whether the BLM framing (and the notion of racial oppression more broadly) can be treated a monolithic category that can be freely transferred from the US context; and what is potentially gain or lost by doing so. Reducing border deaths to the question of BLM, although obviously relevant, may create a false sense of unity and obscure other salient and productive avenues of understanding. This debate in several ways resembles previous criminological discussions about overarching global explanatory frameworks for understanding the use of penal power, for example, neoliberalism (Lacey, 2013). Several scholars have warned against aspirations to universality, the attraction of grand narratives and the tendency to ‘read the emerging—global—landscape too flatly’ at the expense of more grounded empirical explanations (Loader & Sparks, 2002, p. 100; Melossi, 2004; Franko, 2020a).

It is the citizen/alien distinction which guides the rationality of the EU and its member state institutions and has the detrimental effect on official systems of knowledge production and, consequently, also on migrants’ access to rights. Even for an institution such as the European Court of Human Rights, as Marie Bendicte Dembour’s (2015) study shows, human rights tend to take second place to the sovereignty principle. Although important progress has been made on several issues, Dembour (2015, p. 503) suggests that much of migrants’ suffering is left out of account by the court—it is considered illegitimate and outside its area of concern. This, however, is not to say that this suffering does not matter. Instead, the tasks of caring and documenting migrants’ lives are increasingly pushed out of the sovereign states’ sphere of concern and outsourced to various humanitarian actors (Bosworth, 2017; Fassin, 2012). Not only is the task of counting border deaths left to IOM and various research projects but also search and rescue (SAR) missions have been in recent years increasingly taken over by NGOs, which have become the largest single actor in these operations (Carrera & Cortinovis, 2019).

These developments can serve as an illustration of the contemporary hierarchies of citizenship. These hierarchies both have an internal, domestic dimension, as well as external one that entail differentiated values of life within the emerging global order. Although European law unequivocally states as one of its main principles that “everyone’s right to life shall be protected by law”, in practice, this principle becomes a question of operationalization and political will. The lack of political will to protect life at the border, regardless of other objectives, is visible not only in the reluctance of state authorities related to SAR, which has in recent years transformed into active criticism of NGO missions and their increasing criminalization (Franko, 2020b), but is importantly, also reflected in the patterns of knowledge production surrounding border fatalities.

## Studying the Unequal Value of Life

The patterns of protection (or better lack of) migrant lives reveal distinct rationalities that guide state action at political and institutional levels. In contrast to the Foucauldian biopolitics, which is governing the lives of European citizens, political rationalities governing migrant lives have been characterized by theoretical perspectives, which underline the essentially violent nature of sovereignty, and its ability to inflict death and to exclude life from the sphere of legal protection, such as Giorgio Agamben’s (1998) zoëpolitics and Achille Mbembe’s (2003) concept of necropolitics. These perspectives suggest that the “ultimate expression of sovereignty resides, to a large degree, in the power and the capacity to dictate who may live and who must die” (De León, 2015, p. 67). According to Mbembe (2003, p. 16), sovereignty is expressed predominantly as the right to kill; it requires “the strength to violate the prohibition against killing and contrary to subordination that is always rooted in necessity and the alleged need to avoid death, sovereignty definitely calls for the risk of death”.

Criminologists have been in many ways eager to study the structure and operations of state actors and the nature of state sovereignty. Nevertheless, such theoretical perspectives have been based on (Hobbesian and Weberian) assumptions of relative peace and protection of the population. They are more suited for understanding the nature of certain (Western) states, and their relations to their own citizens, less so when it comes to numerous global contexts (Morrison, 2005), where human lives are to a large degree endangered precisely by the actions of state actors (Savelsberg, 2010). The unequal capabilities, and willingness, of states to protect their populations is creating conditions of severe global security inequality, or what Judith Butler (2006, p. 29) describes as the inequitable ‘geopolitical distribution of corporeal vulnerability’.

The recently published *Global Study on Homicide* (UNODC, 2019), for example, reveals dramatic regional variations. While the homicide rate in Europe has declined by 63 per cent since 2002, and is now 3.0 per 100,000 population, in contrast, the homicide rate in the Americas is 17.2 victims per 100,000 population, the highest recorded in the region since reliable records began in 1990. There are though great inter-regional variations.

Excluding all the subregions of Africa, for which complete data are not available, Central America and South America, at 25.9 and 24.2 per 100,000 population, respectively, were the subregions with the highest average homicide rates in 2017, followed by the Caribbean, at 15.1 per 100,000 population. By contrast, the subregions with the lowest levels of homicide, at around 1 victim per 100,000 population per year, were Southern, Western and Northern Europe, East Asia and Oceania (Australia and New Zealand) (UNODC, 2019, p. 11)

The study reveals wide differences between states in terms of their ability to protect their citizens and create security within their territories (Naude et al., 2011). These conditions are inseparably linked with global and neocolonial dynamics where policies developed by Northern countries often threaten the fulfillment of basic collective needs in Southern countries and exacerbate conditions of insecurity for local populations.^3^ A considerable body of scholarship has documented North–South divides as a key driver of environmental conflict and crime, counter-productive drug policies and weaponization of local communities (see inter alia Agozino et al., 2009; Andreas & Nadelmann, 2006; Franko, 2020a, b; Goyes & South, 2016).

Nevertheless, most contemporary and historic theories and grand narratives of modern punishment are based on assumptions about relatively strong statehood as found in the global North (Garland, 2001). These models are, for example, far from the description offered by Henrik Vigh’s (2012, p. 146) illuminating study of Guinea-Bissau, where

the state may have pro forma and de jure existence as a sovereign body, but in terms of both the Weberian and Hobbesian definitions of the state it is effectively obsolete: there is no monopoly on the legitimate use of force and no state to protect or guarantee the security and well-being of its citizens. It is an environment which counters our ‘normal’, hierarchical understanding of the state and our normal idea of political structures.

Consequently, there is a gap in the existing (theoretical and empirical) criminological knowledge, which has yet to rise to the challenge of systematically theorizing issues of crime control and security under conditions of acute state fragility and what Gerlach (2010) termed extremely violent societies (for an exception see Dupont et al., 2003; Braithwaite et al., 2010; Karstedt, 2016). In such contexts, the precarity of human life is often caused by complex global interconnections and defined by a ‘dirty togetherness’ between the state and other organized sources of violence, as shown by Graham Denyer Willis’ (2015, p. 11) study of São Paulo (see also Karstedt, 2013; Rodriguez Ferreira, 2016).

Questions “what kind of state do you live in?” and “what state are you a citizen of?” are crucial for answering how secure you are. Some states are, as Catherine Dauvergne (2008) observes, more sovereign than others. This impacts not only their ability to provide security to their citizens but also to influence the dynamics of international criminal justice which is tilted toward serving the interests of powerful states (Andreas & Nadelmann, 2006; Hurrell, 2007; Franko, 2020a). David Garland’s (2013) encouragement to criminologists to turn attention to the nature of the penal state should, therefore, be extended and include the varieties of statehood found globally. The deeply stratified global order allows citizens of some (Northern) states not only to enjoy high levels of security at home, but is increasingly creating expectations to extend the protection across the world. The growth of global mobility and trade, as well as various types of international military and political intervention, has increased the numbers of those in need of protection in places which are beyond the legal authority of the states they are citizens of (Græger & Lindgren, 2018). Sandvik (2018), for example, shows how, in a court case in Oslo, a Canadian employee of a Norwegian NGO was able to litigate (and win) over his employer arguing a neglect of duty of care during a kidnapping incident in the Dadaab refugee camp in Northern Kenya.

The case also shows that the question “what state are you a citizen of?” is certainly not the only one to ask, since security inequalities are not only determined by state fragility and inability to provide security for their citizens but also on individuals’ purchasing power and possibilities for buying into private security protection (Goold et al., 2010). As a large and productive body of criminological scholarship has shown, private security orders lead to unequal protection and function as ‘club goods’ (Crawford, 2006; Loader & Walker, 2007). These patterns of inequality are particularly pronounced at the global level (O’Reilly, 2015), where private actors such as Control Risks are helping their clients to ‘succeed in a volatile world’.^4^ Not only international businesses but also humanitarian NGOs have become great consumers of private security (Duffield, 2011). However, as Schreter and Harmer (2013) show, for example, in incidents of kidnapping, national NGO staff members suffer greater mortality rates than international staff. In the Oslo court case mentioned above, the Canadian kidnapping victim was able to win a substantial compensation of NOK 5.5 million (around USD $650,000), unlike his Filipino and Kenyan colleagues (Sandvik, 2018).

There are, as Judith Butler (2006, p. 32) succinctly observed in the aftermath of 9/11, ‘radically different ways in which human physical vulnerability is distributed across the globe.’ While certain lives are highly protected, and their loss can mobilize the forces of war, others ‘will not even qualify as “grievable”’ (ibid.). This unequal distribution of corporeal vulnerability, forcefully brought to attention by the BLM movement, is not only a question of geopolitics, but class, racial, gender and other forms of inequality. The geographical demarcations of global North and South are thus an increasingly complicated exercise.

## Which Lives Matter in Criminology?

While questions of the global security inequality offer interesting opportunities to expand the existing criminological frames of understanding, they also show their limitations. The UNODC homicide study, for example, reveals that even though intentional homicide is one of the most measurable and comparable indicators for monitoring violent deaths (UNODC, 2019), it is still undermined by the lack of quality data in some regions and for certain periods. This is particularly the case for certain subregions of Africa. The question of which lives matter in terms of protection and which lives are grievable is, therefore, also a question of which lives are knowable in their loss and vulnerability. Again, the nature of state power and its connections to knowledge (Foucault, 1980) becomes of vital importance and deserves detailed empirical attention. For while the UNODC study reports about the lack of reliable data on homicides in several countries and regions, Heimer (2019, p. 388), for example, observes increasing availability of survey and administrative data and easy access to computing power, which allow for growing sophistication in quantitative analyses of exposure to violence among minority groups in the United States.

Scientific knowledge about the social distribution of corporeal vulnerability, therefore, varies greatly according to the modalities of state power and knowledge. Although this may not always have been the case, in the US context, the modalities of power/knowledge now allow for sophisticated documentation and analyses of various types of victimization. On the other hand, many countries in the global South are still lacking even most basic numbers. In the case of European border deaths, however, we are faced with yet another constellation, where a sophisticated state apparatus with expanding systems of surveillance and analysis (Aas & Gundhus, 2015) is unwilling to employ them and opens for the growing centrality of other, non-state, actors. The vulnerability of life, in this case, is not marked by the absence of knowledge, rather that this is not state produced knowledge. This is by no means an isolated example. NGOs, advocacies, campaigning and philanthropic foundations have become central actors in knowledge production of statistics and knowledge about various forms of transnational crime and victimization (Merry, 2016). However, knowledge produced by these actors differs from traditional academic and state-bureaucratic knowledge production. It focuses on specific forms of crime and victimization and often challenges established standards of scientific objectivity by blurring the boundaries between scientific production and advocay, and reflect preferences and priorities which are often determined by funding.

Although my invitation to turn our analytical attention to global security inequalities rests on an assumption that cross-national comparisons are possible and potentially valuable (Nelken, 2011), it is also mindful of the fact that we need to develop better conceptual and methodological approaches to do so. It should certainly not be read as an invitation to uncritically succumb to what Sally Engle Merry (2016) describes as ‘the seductions of quantification’, which are prevalent in contemporary penchant toward the production of global indicators on issues such as human rights violations, gender violence or sex trafficking. A study of global security inequalities, therefore, needs to take into account that many existing instruments are shaped by global power relations and are inscribed into specific forms of governance. They, in themselves, should make an important object of our scholarly inquiry (Nelken, 2019).

Systems of knowledge production—whether bureaucratic or academic—are inevitably shaped by power relations (Hogg et al., 2017). The struggle for more inclusive academic environments and a more equitable and international criminology is thus faced with a number of challenges. These are not only conceptual, theoretical and methodological, but perhaps even more importantly economic and material, having to do with access to research grants and resources, publishing fire walls, unjust economies of co-authorship, hierarchies of gender, race, age and alike. The dichotomy between developing cross-border connectivity and sharing a common consciousness is maintained by complex material conditions. In imagining alternatives to criminological realities shaped by inequality, we currently lack discourses and ideals of universal citizenship and civil rights which exist on the national level (Western, 2014). However, there might be a lesson to be learnt in Butler’s (2006, p. xiv) attempts to overcome global divisions and divisive power of national self-interest, through an apprehension of our common human vulnerability, ‘one that one cannot will away without ceasing to be human’. Although we cannot deny that vulnerability is globally differentiated, we also share a common human condition, ‘one in which we are, from the start, given over to the other’ (ibid. 31). This, at least, is a start.

## Footnotes

1 My own translation. Source: https://www.nrk.no/vestland/politiet-opplever-mindre-respekt-fra-ungdom-1.15135170.

2 https://frontex.europa.eu/media-centre/news-release/frontex-welcomes-new-standing-corps-recruits-ILr9os.

3 It should be noted though that the lines of demarcation between the global North and South are far from simple. The North–South divide should, as Hogg et al. (2017, p. 5) suggest, ‘be regarded not simply or primarily as a geographical divide so much as a metaphor for power relations’. Many Northern societies contain spaces that can be described as “Southern” and vice versa.

4 Source: https://www.controlrisks.com/.
